# EVALUATION OF EQUITY IN HOSPICE CARE UTILIZATION AMONG MEDICARE-ENROLLED AUTISTIC OLDER ADULTS

**DOI:** 10.1093/geroni/igae098.2171

**Published:** 2024-12-31

**Authors:** Lauren Bishop, Melica Nikahd, J Madison Hyer, Brittany Hand

**Affiliations:** University of Wisconsin Madison, Madison, Wisconsin, United States; The Ohio State University, Columbus, Ohio, United States; The Ohio State University, Columbus, Ohio, United States; The Ohio State University, Columbus, Ohio, United States

## Abstract

Better identification and increased life expectancy of autistic people has given rise to a need to better understand their end-of-life care. The purpose of this study was to compare hospice utilization of autistic older adults to non-autistic older adults using hospice claims from Medicare and examine if known inequities in hospice utilization among the general population are exacerbated among autistic older adults. Our sample included 5,468 autistic older adults and 10,934 matched population comparison older adults who died between 2013-2021. We used multivariable logistic regression to compare these groups on the odds of any, early (at least 28 days before death), intermediate (4-27 days before death), and late (within 3 days of death) hospice utilization. Interaction terms were used to assess whether effects were modified by beneficiary’s sex, race, or rurality. In a sensitivity analysis, we repeated our analyses in unmatched samples. The autistic older adult and matched population comparison groups had similar odds of any hospice utilization (OR: 1.05; 95% CI: 0.98-1.13), early (OR: 1.01; 95% CI: 0.91-1.11), intermediate (OR: 1.03; 95% CI: 0.95-1.13), and late hospice (OR: 1.07; 95% CI: 0.96-1.19). In our models evaluating the impact of other known inequities, we did not find significant interactions between autism diagnostic status and sex, race, or rurality. Results were similar in the unmatched samples. Our findings suggest that autistic older adults experience similar receipt and timing of hospice as non-autistic peers, highlighting the potential power of zero cost, universal health policies in reducing healthcare utilization disparities among autistic people.

